# Effect of Strain Rate, Temperature, Vacancy, and Microcracks on Mechanical Properties of 8-16-4 Graphyne

**DOI:** 10.3390/nano14060556

**Published:** 2024-03-21

**Authors:** Qing Peng, Zeyu Huang, Gen Chen, Yuqiang Zhang, Xiaofan Zhang, Xiao-Jia Chen, Zhongwei Hu

**Affiliations:** 1 School of Science, Harbin Institute of Technology, Shenzhen 518055, China; 2 State Key Laboratory of Nonlinear Mechanics, Institute of Mechanics, Chinese Academy of Sciences, Beijing 100190, China; 22014080032@stu.hqu.edu.cn (Z.H.); 22013080001@stu.hqu.edu.cn (G.C.); 20206251@stu.cqu.edu.cn (X.Z.); 3 Guangdong Aerospace Research Academy, Guangzhou 511458, China; 4 Institute of Manufacturing Engineering, Huaqiao University, Xiamen 361021, China; 19013080047@stu.hqu.edu.cn; 5 Institute of Mechanical Engineering and Automation, Huaqiao University, Xiamen 361021, China; 6 College of Aerospace Engineering, Chongqing University, Chongqing 400044, China

**Keywords:** 8-16-4 graphyne, mechanical properties, molecular dynamics, vacancy defect, microcrack

## Abstract

The 8-16-4 graphyne, a recently identified two-dimensional carbon allotrope, exhibits distinctive mechanical and electrical properties, making it a candidate material for flexible electronic applications. This study endeavors to enhance our comprehension of the fracture behavior and mechanical properties of 8-16-4 graphyne. The mechanical properties of 8-16-4 graphyne were evaluated through molecular dynamics simulations, examining the impact of boundary conditions, temperature, and strain rate, as well as the coupled interactions between temperature, vacancy defects, and microcracks. The findings reveal that 8-16-4 graphyne undergoes fracture via the cleavage of ethylene bonds at a critical strain value of approximately 0.29. Variations in boundary conditions and strain rate influence the fidelity of tensile simulation outcomes. Temperature, vacancy concentration, and the presence of microcracks markedly affect the mechanical properties of 8-16-4 graphyne. In contrast to other carbon allotropes, 8-16-4 graphyne exhibits a diminished sensitivity to vacancy defects in its mechanical performance. However, carbon vacancies at particular sites are more prone to initiating cracks. Furthermore, pre-existing microcracks within the material can potentially alter the fracture mode.

## 1. Introduction

In the realm of all-carbon chemistry, the introduction of acetylene bonds markedly diversifies carbon isomers, encompassing graphyne and its derivatives [1,2,3,4,5]. Acetylene groups can lower bonding energy and modulate optical, thermal, and electronic properties in various ways [6,7,8,9]. These materials also manifest exceptional elasticity, adjustable Poisson’s ratio [10], high fracture toughness, and impressive strength. A comprehensive understanding of the mechanical properties of carbon allotropes is crucial for their effective utilization. Mechanical properties of graphene and other carbon allotropes have been extensively explored by researchers [11,12,13,14,15,16,17,18]. Lee et al. [19] utilized nanoindentation to measure the failure strength and Young’s modulus of single-layer graphene, obtaining values of 42 Nm^−1^ and 1.0 TPa, respectively. Zhang et al. [20] investigated the impact of pre-existing cracks on the fracture toughness of graphene samples through in-situ tensile experiments. Feng et al. [21] characterized crack progression and fracture toughness anisotropy in single-crystalline graphene monolayers via in-situ tensile tests and modeling.

However, conducting mechanical testing on two-dimensional carbon materials through experimental means is challenging due to limitations and errors in material preparation and experimental methods. Consequently, an increasing number of researchers are turning to numerical simulation methods to investigate the mechanical properties of carbon allotropes [22,23,24,25,26,27,28,29,30,31,32,33,34,35]. Peng et al. [36] employed first-principles calculations to predict the impact of pressure on graphyne’s second-order elastic constants, in-plane Young’s modulus, and Poisson’s ratio. Zhang et al. [37] studied the mechanical properties and failure mechanisms of four different types of graphene using molecular dynamics. Their results indicated a considerable reduction in failure stress and Young’s modulus in the presence of acetylene bonds, and this reduction was found to be directly proportional to the percentage of bonds. Xie et al. [38] investigated the mechanical properties of PSI-graphene under varying tensile angles. Akhunova et al. [39] investigated the fracture and mechanical properties of defective and defect-free rippled graphene. The results indicate that wrinkling significantly reduces the ultimate tensile strength and Young’s modulus of graphene, while increasing the fracture strain.

The graphyne structure, generated through techniques like mechanical exfoliation and chemical vapor deposition (CVD), inevitably harbors defects [40]. Numerous experimental and atomic simulation studies on defective graphyne have demonstrated that defects exert a substantial influence on the mechanical properties of two-dimensional materials [41,42,43,44,45,46,47,48]. Sun et al. [49] investigated the impact of vacancy defects on the mechanical characteristics of graphene, revealing that vacancy defects induce stress concentration, leading to a reduction in failure strength by approximately 17.7% due to a single vacancy. Yu et al. [50] conducted a comparative analysis on the influence of geometric shape and orientation of cracks on the mechanical properties of graphene. Yin et al. [51] explored the applicability of the Griffith criterion, designed for continuous medium theory, in discrete atomic systems. Their findings indicate that the Griffith criterion remains effective for cracks as small as 10 nm, but it might overestimate the strength of shorter cracks due to local effects at the crack tip. Alahmed et al. [52] explored the influence of the crack angle on the mechanical properties of single-layer graphene. Their findings revealed that the crack front with a vertex angle of 60° corresponds to the weakest configuration, exhibiting the lowest fracture toughness and fracture strain.

The allotrope 8-16-4 graphyne, a recently proposed single-atom-thick carbon allotrope [53], derives its name from the distinctive structural feature of four carbon atoms interconnected in an octagonal shape, forming a 16-membered ring. Bandyopadhyay et al. [54] comprehensively validated the dynamical, mechanical, and thermodynamic stability of 8-16-4 graphyne through first-principles calculations. They discussed in detail its synthetic feasibility and proposed a potential chemical synthesis pathway. Jafari et al. [55] investigated the adsorption behavior of transition metal atoms on 8-16-4 graphyne using DFT-D2. The results indicate that 8-16-4 graphyne exhibits high adsorption capability and characteristics preventing the aggregation of transition metal atoms, making it a potential candidate for single-atom catalyst carriers and hydrogen storage materials. Tromer et al. [53] conducted a comprehensive investigation into its mechanical, structural, optical, and electrical properties using first-principles calculations. Their findings revealed a unique characteristic of 8-16-4 graphyne: its electrical properties remain unchanged under moderate strains, rendering it a promising candidate for applications in flexible electronic devices. 

In order to further investigate the potential applications of 8-16-4 graphyne in these domains, a comprehensive understanding of its mechanical properties is essential. This study employs MD simulations to explore the mechanical performance of 8-16-4 graphyne under various environmental conditions, including the influence of boundary conditions, temperature, and strain rate on the material’s plasticity and fracture response. Additionally, a comparative analysis of the mechanical behavior of defective and defect-free models of 8-16-4 graphyne is conducted, along with an examination of the coupling effects of temperature and defects. 

## 2. Materials and Methods

**Figure 1:**
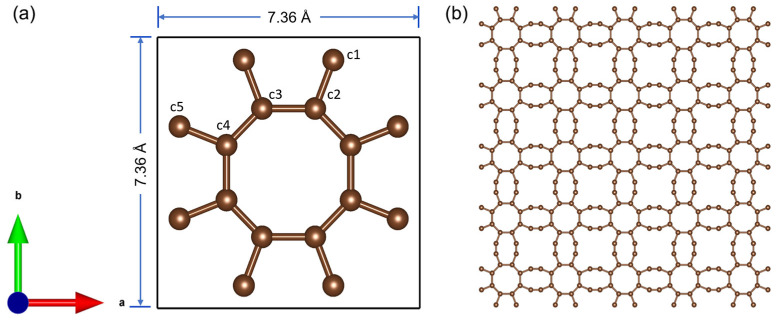
Schematic diagram of the 8-16-4 graphyne structure. (**a**) Single-cell structure, (**b**) a monolayer of pristine graphyne was produced by VESTA. The letters “a” and “b” in the bottom-left corner represent the orientation of the coordinate system.

The 8-16-4 graphyne model shown in Figure 1 has a structure composed of octagonal and 16-membered carbon rings [56]. First-principles calculations indicate that this structure exhibits both dynamic and thermal stability.

To investigate the mechanical behavior of 8-16-4 graphyne, we performed uniaxial tension simulations using the Large-scale Atomic/Molecular Massively Parallel Simulator (LAMMPS 23-June-2022) [57]. Considering that bond breakage and formation occur during fracture processes, we selected the adaptive intermolecular reactive bond order potential (AIREBO-M) [58]. The AIREBO potential is considered the most accurate interatomic potential for modeling SLGS mechanical, fracture, and thermal properties [59], The AIEBO-M potential is a modified form of the AIREBO potential and exhibits more accurate performance in high-pressure systems.

In molecular dynamics (MD) simulations, all models are square-shaped. Except for the second section, periodic boundary conditions are applied in the x and y directions, and free boundary conditions are applied in the out-of-plane z direction, with a 50 Å vacuum layer. Convergence tests were conducted on six groups of simulation systems with different sizes ranging from 1440 to 19,600 atoms. The results indicate that, for defect-free systems, the system size has a negligible impact on the test results. Therefore, a simulation system with 3600 atoms was adopted for defect-free system calculations. The Verlet integration method was employed to numerically integrate the equations of motion for atoms over time with a time step of 0.1 fs. The initial structure of 8-16-4 graphyne was optimized using the conjugate gradient method. During temperature initialization, initial velocities were assigned only in the x and y directions, preventing the influence of wrinkles in the relaxation phase on the tensile test results. Subsequently, a 4 ps relaxation was conducted in the NPT ensemble to eliminate internal stresses and achieve a dynamically stable state. Except for Section 3.1, all simulated strain rates were set at 1 × 10⁹/s engineering strain/second. Material stretching was achieved by increasing the periodic simulation box size along the loading direction, and atomic positions were remapped every time the simulation box size was changed. The tensile stress in the x-direction is calculated by evaluating the stress within the entire simulated box’s yz plane, multiplying it by the length of the box along the *z*-axis, and then dividing the result by 3.34 Å (a commonly used effective thickness for two-dimensional carbon materials). Stress and strain were sampled at every time step, with the averages outputted every 250 steps. OVITO 3.9.4 was used for data post-processing and visualization [60]. In the visualized images, atomic colors represent von Mises stress values.

The analysis of experimental results primarily focuses on “failure strength”, “fracture strain”, “Young’s modulus”, and “toughness”, all of which are extracted from the stress-strain curve. The failure strength is the maximum value on the stress-strain curve. The fracture strain is the corresponding strain value at this stress. Young’s modulus is the slope of the linear elastic region in the stress-strain curve within the initial 4% engineering strain. Toughness is the energy absorbed by the material before reaching the fracture strain, obtained by integrating the curve.

## 3. Results and Discussions

### 3.1. Boundary Effect

**Figure 2:**
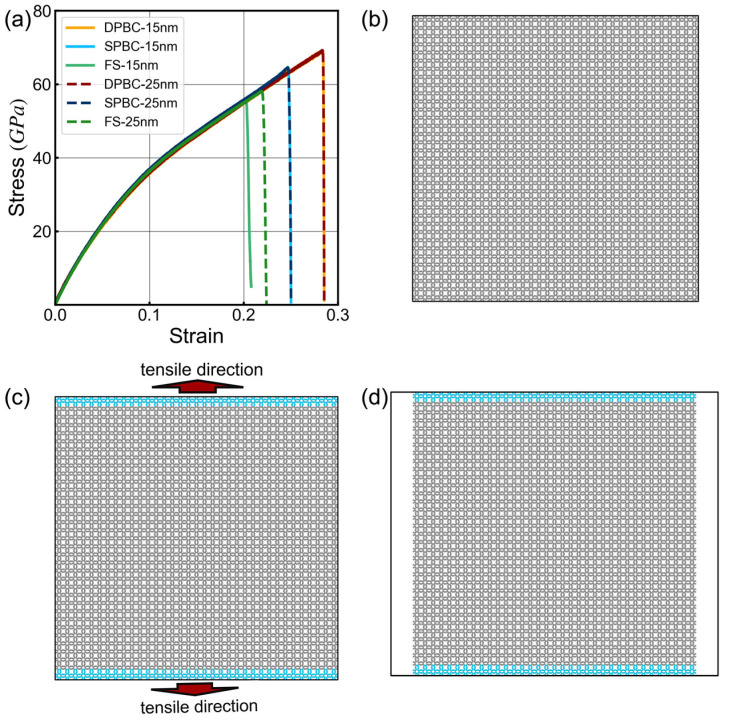
Tensile test results of the 8-16-4 graphyne model under different boundary conditions. The blue atoms are fixed in the simulation to break the periodicity. (**a**) Stress-strain curves; (**b**) Double periodic boundary condition model (DPBC); (**c**) Single periodic boundary condition model (SPBC); (**d**) Finite-size model (FS).

In MD simulations, the system size is typically confined to the scale of a few hundred nanometers, much smaller than the scales employed in experiments. Therefore, periodic boundary conditions (PBC) are commonly employed to simulate the boundary effects arising from this substantial size difference, making the system resemble an infinite one. However, this assumption of a perfectly infinite repeating unit approximation may introduce additional errors, such as the suppression of long-range correlations, and the complete neglect of edge effects, which could lead to an overestimation of the material’s mechanical properties. Consequently, we conducted uniaxial tensile simulations on 8-16-4 graphyne under different boundary conditions and model sizes to examine the influence of boundary conditions on the simulations (Figure 2b–d).

The calculated Young’s modulus of 8-16-4 graphyne is nearly identical under different boundary conditions, but there are significant differences in fracture strain and failure strength (Figure 2a). Under periodic boundary conditions, the material exhibits higher failure strength. The failure strength calculated for a 15 nm side length model is 13.5% and 19.3% lower than that of the DPBC under SPBC and FS, respectively. Similarly, the 25 nm side length model shows a 10.1% and 16.1% lower failure strength under SPBC and FS compared to the DPBC model. As the system size increases, the results of the finite-size model approach closer to those obtained using periodic boundary conditions. The selection of boundary conditions involves a complex competition mechanism between errors arising from neglecting edge effects and those resulting from size differences. In general, models using periodic boundary conditions tend to provide results closer to experimental outcomes [61].

### 3.2. Strain Rate Effect

Due to the temporal constraints in MD simulations, the strain rates used are frequently orders of magnitude higher than those applied in experiments. This significant difference may result in non-equilibrium effects, where, at excessively high strain rates, the model may not fully relax, leading to an inaccurate representation of the material’s actual behavior. Furthermore, the strain rate can influence the deformation mechanisms of materials; in simulations, materials require sufficient time to respond to tensile loads. At high strain rates, certain behaviors conducive to deformation or fracture, such as dislocation motion, initiation and propagation of cracks, etc., may not fully develop as they would at low strain rates. Therefore, it is imperative to choose an appropriate strain rate for tensile simulations to strike a balance between accuracy and computational efficiency. To this end, we investigated the effects of five strain rates ranging from 5 × 10^8^/s to 5 × 10^10^/s on tensile tests. This is a common strain rate range in the simulation of stretching for two-dimensional carbon materials [62,63].

**Figure 3:**
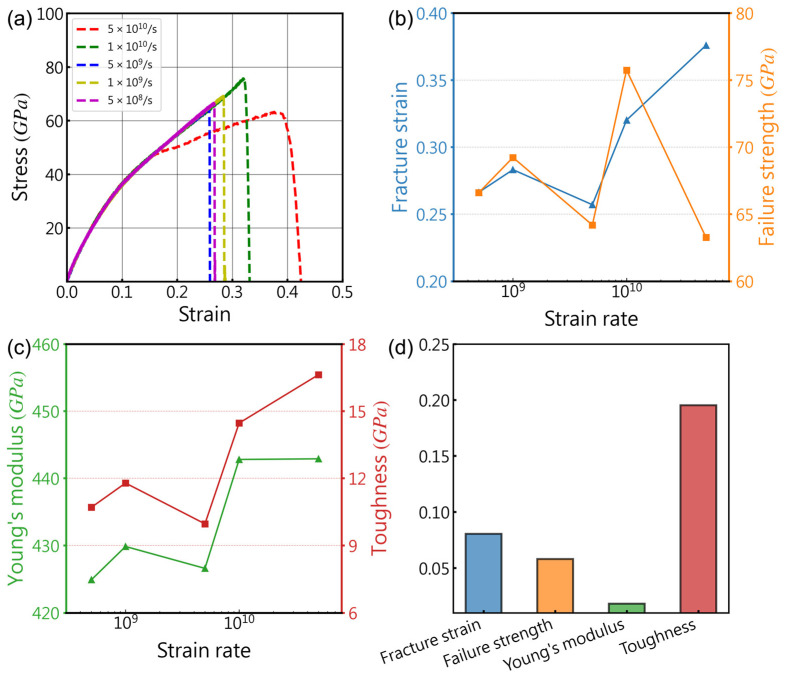
Uniaxial tension tests were performed on 8-16-4 graphyne at different strain rates. (**a**) Stress-strain curves, (**b**,**c**) Relationship between main mechanical properties and strain rate, (**d**) Percentage variance of mechanical properties.

Figure 3 displays the results of MD tensile simulations conducted at different strain rates spanning three orders of magnitude. It can be observed that the impact of strain rate on the four mechanical properties obtained from the tests exhibits a non-monotonic trend, which is particularly evident in the fluctuating variations depicted in Figure 3b for fracture toughness and failure strength. These fluctuations suggest that the influence of strain rate on mechanical property testing may involve multiple competing mechanisms influencing material response. As shown in Figure 3c, the overall trend of the material’s Young’s modulus slightly increases with the rise in strain rate. This may be attributed to artifact resulting from the inability of the model to fully relax in high strain rate simulations. Therefore, in subsequent simulations, we opted for a strain rate of 1 × 10^9^/s, as it resulted in mechanical performance parameters with errors within 5% compared to lower strain rates.

In comparison with other two-dimensional materials like graphene, the strain rate sensitivity of the mechanical properties of 8-16-4 graphyne is more pronounced [64] but not as significant as observed in materials such as metal nanomaterials or carbon nanotubes [65,66]. Figure 3d presents the variance of the four mechanical properties obtained from five tests at different strain rates, with variance values of 0.15 and 0.065 for fracture strain and failure strength, respectively, and only 0.016 for Young’s modulus. This indicates that, compared to factors such as temperature and defects, the influence of strain rate on the mechanical properties of 8-16-4 graphyne is relatively small, and the strain rate primarily affects the large strain regions in the simulations. This trend is consistent with similar studies on other two-dimensional carbon materials [67], demonstrating the strain rate stability of the mechanical properties of 8-16-4 graphyne.

### 3.3. Temperature Effect

**Figure 4:**
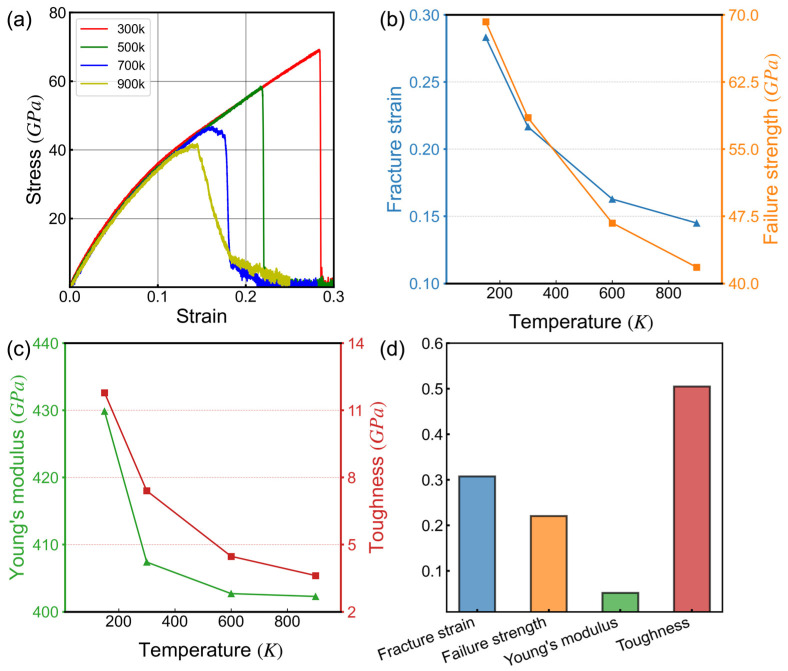
Uniaxial tension tests were performed on 8-16-4 graphyne at different temperatures. (**a**) Stress-strain curves; (**b**,**c**) Relationship between main mechanical properties and temperature; (**d**) Percentage variance of mechanical properties.

Temperature is a significant factor influencing the mechanical properties of materials [68]. To ensure the reliable application of two-dimensional materials in extreme temperature environments, a detailed investigation of the correlation between their mechanical properties and temperature is crucial. Figure 4 presents the stress-strain curves of 8-16-4 graphyne at various temperatures and illustrates how Young’s modulus, failure strength, fracture strain, and fracture toughness change with temperature.

At 300 K, 8-16-4 graphyne exhibits a Young’s modulus of approximately 438 GPa and a fracture strain of 0.29, which are approximately 40% and 179% of those of graphene, respectively. As the temperature increases, the material gradually softens. When the temperature reaches 900 K, the Young’s modulus and failure strength of 8-16-4 graphyne decrease by approximately 7% and 41%, respectively, compared to their values at 300 K. Fracture strain and fracture toughness also exhibit a decreasing trend with increasing temperature, with the slope decreasing as the temperature rises. In contrast, the temperature dependence of the mechanical properties of graphene is nearly linear [69]. 

Furthermore, at temperatures of 500 K and below, the fracture of 8-16-4 graphyne is entirely brittle, as there is no apparent yielding phenomenon observed in the stress-strain curve at the fracture location. However, as the system temperature increases to 700 K, the material exhibits some toughness. The strain corresponding to the maximum stress is less than the strain at complete failure, and this phenomenon becomes more pronounced at 900 K.

**Figure 5:**
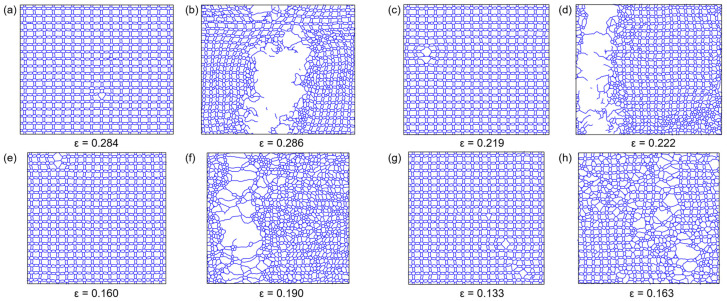
The atomic motion trajectories during tensile fracture at different temperatures, utilizing OVITO’s bond generation function to connect atoms with distances less than 2.0 Å. (**a**,**b**) 300 K. (**c**,**d**) 500 K. (**e**,**f**) 700 K. (**g**,**h**) 900 K.

This phenomenon may be related to structural transformations occurring at higher temperatures and under tensile strain. Figure 5 displays atomic trajectory snapshots of 8-16-4 graphyne at the initiation of bond breaking and complete failure, captured at two time points, under temperatures of 300 K, 500 K, 700 K, and 900 K. It can be observed that at 300 K and 500 K, the crack rapidly propagates after the ethylene bond breaks. However, at 700 K, a portion of long carbon chains, known as carbyne, is formed among the disrupted structures. Carbyne is considered to possess superior mechanical properties compared with two-dimensional carbon structures [70,71]. Zhu et al. [72] conducted a detailed investigation of this phenomenon through MD simulations, proposing that the size of carbon rings and temperature are crucial factors leading to the formation of carbyne in the tensile deformation of carbon structures. The formation of one-dimensional carbon chains is the reason for the plastic behavior observed in the stress-strain curve of 8-16-4 graphyne at elevated temperatures. Additionally, at higher temperatures, post-failure evolution of 8-16-4 graphyne results in the formation of new structures, including 5-membered and 6-membered rings. At 900 K, the defect generated by the first ethylene bond breaking does not rapidly propagate to form a crack; instead, cracks nucleate at multiple locations. The material took a longer time to progress from the initiation of bond breaking to complete failure.

### 3.4. The Vacancy-Defect Effect

Vacancy defects are the most common imperfections in crystalline materials. In two-dimensional carbon structures, factors such as material preparation and high-energy particle impacts can introduce vacancy defects into the material [73,74]. These vacancy defects can be employed to tailor various material properties to meet specific application requirements, while simultaneously exerting an influence on the material’s mechanical performance [75,76]. To investigate the impact of vacancies on the mechanical properties of 8-16-4 graphyne, we randomly deleted 0.1%, 0.3%, 0.5%, 1%, 3% and 5% of atoms in a system containing 10,000 carbon atoms to create vacancy defects. Subsequently, we stretched these models at different temperatures to investigate the coupled effects of temperature and defects on the mechanical properties of 8-16-4 graphyne.

**Figure 6:**
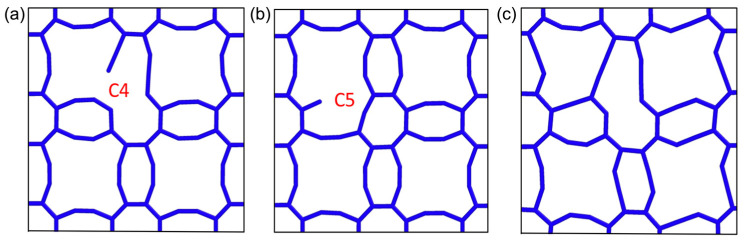
Uniaxial tensile tests on models with vacancy defects. (**a**,**b**) Two types of inequivalent vacancy defect sites. (**c**) Bonding during equilibrium or stretching processes. C4 and C5 respectively represent different vacancy defect for sp^2^ and sp hybridized sites.

In 8-16-4 graphyne, there are two types of carbon atoms with distinct sp and sp^2^ hybridization structures. After the molecular statics structure optimization, the atomic structures near these vacancy defects are shown in Figure 6a,b respectively. It can be observed that the sp carbon atom vacancy (C5) leads to significant deformation in the surrounding octagon ring.

Randomly generated defects include vacancies at both of these different sites. However, upon reaching the fracture limit, cracks consistently initiate from the vacancy defects at the C2 or C3 positions (Figure 1). This suggests that, during stretching along the *x*-axis, the vacancy defects at this particular site are more prone to inducing crack initiation compared to other sites.

**Figure 7:**
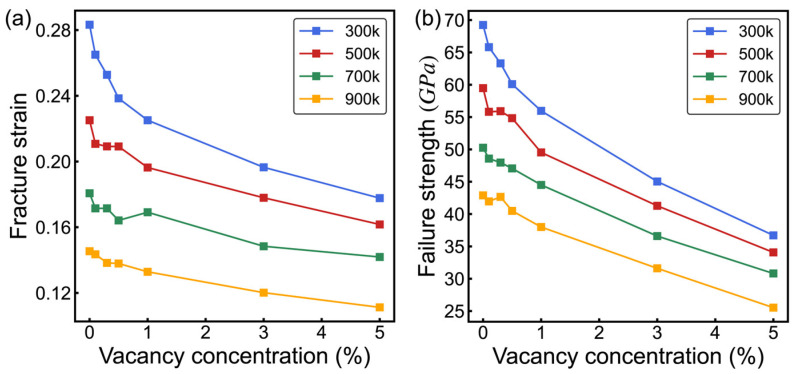
The coupling effect of temperature and vacancy defects on the mechanical properties of 8-16-4 graphyne. (**a**) Fracture strain. (**b**) Failure stress.

Figure 7 illustrates the coupled effects of temperature and vacancy defects on the mechanical properties of 8-16-4 graphyne. As the vacancy defect concentration increases, both the material’s fracture strain and failure strength significantly decrease. It is noteworthy that, compared to other two-dimensional carbon materials, 8-16-4 graphyne exhibits lower sensitivity to vacancy defects. Compared to the defect-free system, the failure strength of 8-16-4 graphyne with a 1% vacancy concentration decreases by 19%. In contrast, graphene with the same proportion of vacancy defects experiences a 31% reduction in failure strength [38]. This characteristic may be attributed to the longer carbon chains in the structure of 8-16-4 graphyne, allowing the freely dangling carbon chains generated by vacancy defects to form new bonds with other carbon atoms during the stretching process. Figure 6c illustrates the structure near the vacancy defects during the stretching process, showing the formation of new chemical bonds. As the temperature increases from 300 K to 900 K, compared to the defect-free model, the models with a 5% vacancy concentration exhibit a reduction in fracture strain of 0.106, 0.064, 0.038, and 0.032, and a corresponding decrease in failure strength of 32.5 GPa, 25.5 GPa, 19.4 GPa, and 17.4 GPa, respectively. Elevated temperatures enhance atomic thermal vibrations, making suspended atoms more prone to bond with other carbon atoms during the stretching process, thereby compensating for the detrimental impact of defects on the mechanical robustness of 8-16-4 graphyne.

Additionally, the actual position and distribution of defects within the material lattice may not perfectly align with the random atomic removal simulated in our study. This disparity could lead to differences in the material’s response to vacancy defects, presenting a challenge for experimental validation.

### 3.5. Cracks and Fracture Toughness

Pre-existing cracks exert a substantial impact on the mechanical properties of materials. In practical scenarios, material fracture frequently initiates from the propagation of pre-existing microcrack tips rather than originating from the bonds between atoms in perfect crystals. As a result, the failure strength of a material is frequently intricately connected to the presence of pre-existing cracks.

According to Griffith’s formula [77]:

(1) $$\sigma_{f}=\sqrt{\frac{2E\gamma_{s}}{\pi a}}$$

The surface energy ($\gamma_{s\;}$) and crack radius (*a*) exert a significant influence on the tensile failure strength. When the stress concentration at the crack tip attains the energy required to break a bond or a specific number of bonds, crack propagation initiates. Furthermore, the fracture behavior is influenced by the distinct atomic bonding at the crack tip, and temperature also significantly affects the initiation and propagation of cracks in the material. Consequently, we conducted an investigation into the mechanical properties of 8-16-4 graphyne under the influence of cracks and temperature. Our model involved the introduction of cracks with varying lengths. The crack lengths were set at 24, 26, 31, 33, 38, 41, 46, and 56 Å, respectively. The irregular crack length settings are based on considerations of the discrete nature of atomic structure.

**Figure 8:**
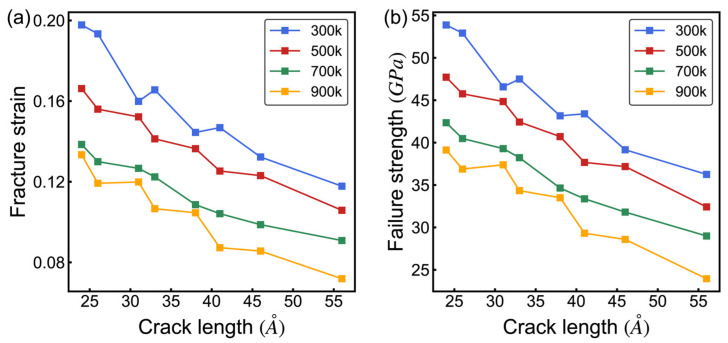
Coupled influence of temperature and crack length on the mechanical properties of 8-16-4 graphyne. (**a**) Fracture strain. (**b**) Failure strength.

**Figure 9:**
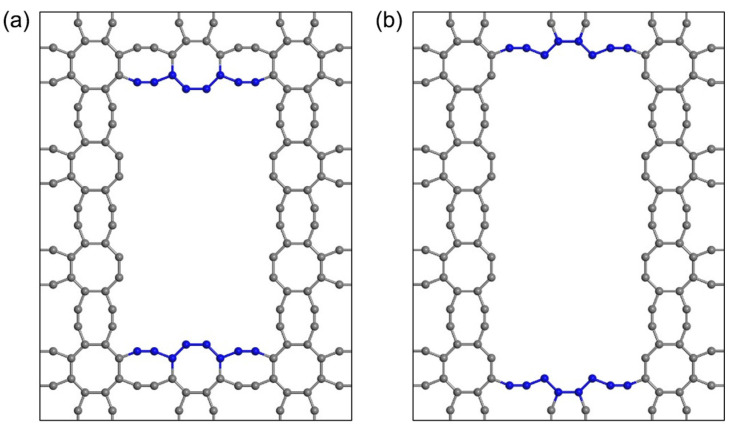
8-16-4 graphyne with cracks that cut off at different locations with different termination. (**a**) Crack with a-type termination. (**b**) Crack with b-type termination. The atoms at the crack tip are highlighted in blue.

Figure 8 illustrates the coupled effects of temperature and varying crack lengths on the mechanical properties of 8-16-4 graphyne. Longer cracks weaken the material’s mechanical performance more significantly, with both fracture strain and failure strength decreasing as the crack length and temperature increase. The relationship between the mechanical properties of the material and the crack length is not linear but exhibits a fluctuating, monotonically decreasing trend. This behavior is attributed to the different atomic bonding configurations at the crack tips. Figure 9 shows two crack models with different lengths and distinct crack-tip configurations. This difference results in variations in the initiation of bond fracture under failure stress. Therefore, cracks with these two different tips exhibit inconsistent degrees of material weakening, with different trends and influencing mechanisms at varying temperatures.

**Figure 10:**
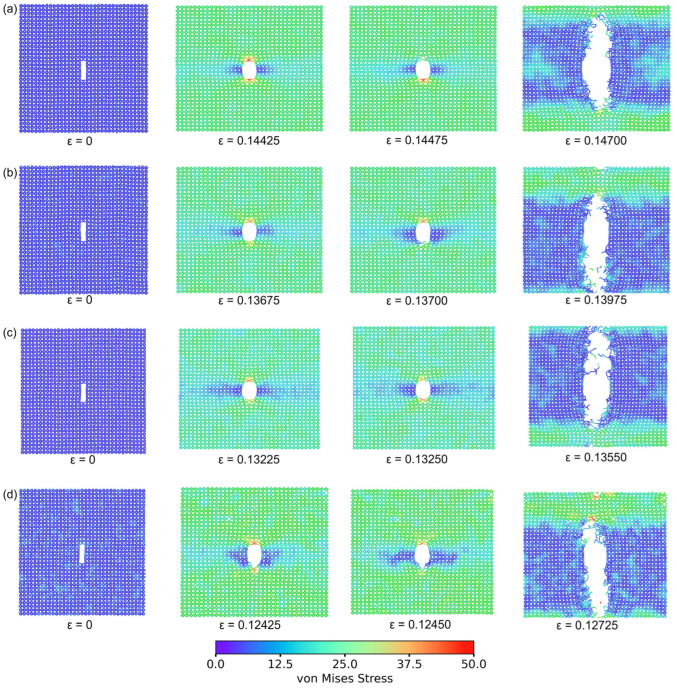
Fracture morphology of the 8-16-4 graphyne model with cracks at different temperatures. (**a**) 300 K. (**b**) 500 K. (**c**) 700 K. (**d**) 900 K.

Subsequently, the deformation and fracture behavior of the 8-16-4 graphyne structure under uniaxial tension with different crack tips were analyzed through atomic trajectory visualization (Figure 10). Under tension along the *x*-axis, chemical bonds parallel to the *x*-axis experience higher stress compared to bonds oriented perpendicular to the *x*-axis. This behavior can be ascribed to the cross-shaped structure inherent in 8-16-4 graphyne. Due to the inherent weakness of double bonds compared to triple bonds, the ethylene double bonds parallel to the *x*-axis began to fracture during further deformation, becoming the focal point for crack initiation. As shown in Figure 5a, in the defect-free model, the fracture of the C2-C3 ethylene bond induced the initiation of the crack. Subsequently, the octagon was destroyed, and the crack propagated along the *y*-axis.

Furthermore, as described in Section 3.3, at temperatures below 500 K, following the initial bond breakage in the system, local defects rapidly develop, forming cracks. Subsequently, the cracks rapidly propagate along the direction perpendicular to the stretching axis, further resulting in complete material fracture. In defect-free systems at higher temperatures, due to lower stress in the system, local chemical bond fractures are less likely to develop into cracks, and defects occur at multiple positions in the model (Figure 5h). However, pre-existing microcracks in the system may contribute to variations in material failure and fracture mechanisms. In the crack model at 900K, despite the presence of some sp carbon chains and defects sprouting from multiple locations, the overall fracture mechanism of the material is more similar to that at low temperatures. The initial defects rapidly propagate from the pre-existing crack tip, and the fracture process no longer exhibits significant yielding phenomena (Figure 10d).

## 4. Conclusions

This study employs MD simulations to investigate the influence of boundary conditions, strain rate, temperature, vacancy defects, and cracks on the mechanical properties of 8-16-4 graphyne. Our research results indicate that models with periodic boundary conditions calculate higher mechanical properties for the material compared to finite-size models. There is no consistent correlation between strain rate and the tensile mechanical properties of the material. However, excessively high strain rates can induce non-equilibrium effects, leading to the overestimation of Young’s modulus at high strain states and inaccurate estimates of failure strength and fracture strain. Temperature significantly impacts the material’s performance, with the Young’s modulus decreasing by 7% and failure stress decreasing by approximately 41% as the temperature rises from 300 K to 900 K. Elevated temperatures influence the fracture mode of 8-16-4 graphyne, inducing some yielding behavior during large deformation stages. Furthermore, compared to similar carbon materials, 8-16-4 graphyne exhibits a relatively low sensitivity to vacancy defects. This behavior is attributed to its inherent longer carbon chain structure, allowing unsaturated carbon atoms at defect sites to form new chemical bonds under tension, compensating for the loss in strength. The sensitivity of 8-16-4 graphyne to vacancy defects further decreases with increasing temperature. Lastly, the study demonstrates that the presence of cracks significantly degrades the mechanical performance of 8-16-4 graphyne, and models with pre-existing cracks tend to exhibit brittle fracture even at high temperatures. Our results provide atomic-scale insights into the mechanical behavior of 8-16-4 graphyne under various parameters, offering valuable information for the design and application of this material in flexible electronic devices.

## Data Availability

The datasets generated during and/or analyzed during the current study are available from the corresponding author on reasonable request.
